# Associations Between Cereal-Based Food Consumption and Urinary Mycotoxin Biomarkers in a Hungarian Population

**DOI:** 10.3390/toxins18100436

**Published:** 2026-10-10

**Authors:** Tamás Schieszl, György Kövér, Lucia Gambacorta, Michele Solfrizzo, Lajos Biró, Györgyi Arató, Andrea Zentai, Brigitta Bóta, András Szabó, Melinda Kovács

**Affiliations:** 1 HUN-REN-MATE, Mycotoxins in the Food Chain Research Group, Science, Guba S. Str. 40, 7400 Kaposvár, Hungary; 2 MATE Doctoral School of Agricultural and Food Sciences, Guba S. Str. 40, 7400 Kaposvár, Hungary; 3 Institute of Animal Sciences, Kaposvár Campus, Hungarian University of Agricultural and Life Sciences, Guba S. Str. 40, 7400 Kaposvár, Hungary; 4 Institute of Sciences of Food Production (ISPA), National Research Council (CNR), 70126 Bari, Italy; 5 Department of Dietetics and Nutritional Sciences, Faculty of Health Sciences, Semmelweis University, 1088 Budapest, Hungary; 6 NutriComp Ltd., Üllői Street 179, 1091 Budapest, Hungary; 7 Department of Digital Food Science, Institute of Food Chain Science, University of Veterinary Medicine Budapest, István Street 2, 1078 Budapest, Hungary; 8 Agro-Biotechnology and Precision Breeding for Food Security National Laboratory, Institute of Physiology and Nutrition, Kaposvár Campus, Hungarian University of Agricultural and Life Sciences, Guba S. Str. 40, 7400 Kaposvár, Hungary

**Keywords:** biomonitoring, co-occurrence, dietary exposure, urine, biomarker, fumonisins, DON, ZEN, OTA

## Abstract

Mycotoxins are common contaminants of the food and feed chain and have been associated with developmental disorders, hepatotoxicity, nephrotoxicity, and various types of cancer. This study aimed to estimate dietary mycotoxin exposure in the Hungarian population and to identify the major cereal-based food contributors. Urinary mycotoxin concentrations were measured by LC–MS/MS, and population exposure was assessed using estimated daily intake (EDI) values. Associations between food consumption and urinary mycotoxin levels were evaluated using Spearman correlation analysis on urine samples collected in year 2018 and 2020. The average EDI values were below the tolerable daily intake (TDI) for all mycotoxins in both years, except for ochratoxin A in 2018. Distinct exposure patterns were observed between the two sampling years. In 2018, consumption of oats and oat-based products showed a moderate positive correlation with urinary fumonisin B_2_ levels (r_(s)_ = 0.497, adjusted *p* = 0.001962350574942). In 2020, wheat and wheat-based product consumption correlated with urinary deoxynivalenol levels (r_(s)_ = 0.6868, adjusted *p* = 0.009509496), while corn and corn-based product consumption correlated with urinary zearalenone levels (r_(s)_ = 0.6137, adjusted *p* = 0.04134226737). These findings highlight differences in dietary mycotoxin exposure between the studied populations and sampling periods and emphasize the importance of human biomonitoring to identify major dietary mycotoxin sources/carriers and support exposure and risk assessment.

## 1. Introduction

Mycotoxins—secondary metabolites produced by filamentous fungi—pose a significant risk to human health due to their widespread occurrence in the food chain. They are implicated in the development of numerous diseases, including hepatopathies and nephropathies, cancer, immunosuppression, and genotoxic effects, among others [1]. Human exposure to mycotoxins occurs predominantly through the consumption of contaminated foodstuffs. Among the most relevant mycotoxins are aflatoxins, ochratoxin A (OTA), and *Fusarium* toxins, particularly deoxynivalenol (DON), zearalenone (ZEN), and fumonisins (FUM) [2]. Over the past three years, the occurrence of major mycotoxins in finished feed samples from the Central European region has shown the following trends. In 2023, DON (63%), ZEN (48%), and FUM (48%) were the most frequently detected mycotoxins [3]. In 2024, their prevalence further increased (DON: 76%, ZEN: 58%, FUM: 55%) [4] while in 2025 type B trichothecenes (including DON and nivalenol (NIV)) remained the most prevalent group (77%), followed by ZEN (53%) and FUM (52%) [5].

Risk assessment constitutes the foundation of modern food safety systems, with exposure assessment representing its first and essential step. Exposure assessment aims to estimate the human intake of hazardous substances by quantifying their occurrence in food or biological matrices in relation to the toxicological properties. Exposure assessment is defined as the qualitative and/or quantitative evaluation of the likely intake of biological, chemical, or physical agents via food, as well as from other relevant exposure sources [6].

For the above-mentioned major mycotoxins, the Joint FAO/WHO Expert Committee on Food Additives (JECFA), a scientific advisory body of the World Health Organization (WHO) and the Food and Agriculture Organization (FAO), has established health-based guidance values, such as evaluated their hazards in several sessions and established tolerable intake values (e.g., tolerable daily intake, TDI), which form the basis for regulatory limits [7].

Human exposure to mycotoxins can be estimated by combining food consumption data with measured contamination levels. In Hungary, previous studies have shown that the probable daily intake (PDI) of fumonisin B_1_ (FB_1_) and fumonisin B_2_ (FB_2_) was substantially below the TDI established by JECFA and EFSA. Nevertheless, in approximately 1% of individuals, the estimated PDI exceeded the EFSA TDI of 1 μg/kg body weight introduced in 2018 [8]. While food contamination and consumption data provide a useful basis for population-level exposure assessment, they do not account for potential non-dietary exposure routes and are therefore limited in their applicability for individual-level risk characterization. Moreover, such approaches are inherently constrained by uncertainties related to dietary reporting and sampling representativeness. Biomarker-based approach can partially overcome these limitations by enabling the assessment of internal exposure and additionally allows for the evaluation of combined exposure to multiple mycotoxins. Urine is the most commonly used biological matrix for mycotoxin biomonitoring, followed by blood (plasma and serum), feces, and, less frequently, breast milk. In the absence of comprehensive human toxicokinetic data, exposure estimation based on human biomarkers often relies on toxicokinetic models and parameters derived from experimental animal studies, introducing additional uncertainty [9]. Nevertheless, urinary biomarker analysis has been shown to represent a reliable and efficient approach for assessing exposure to a broad range of mycotoxins, including DON, FB_1_, OTA, ZEN, α-zearalenol (α-ZOL), β-zearalenol (β-ZOL), and aflatoxin M_1_ (AFM_1_). This methodology is particularly advantageous in contexts where food sample availability is limited and dietary intake data are incomplete or uncertain [10].

Despite these advances, exposure estimates obtained from different assessment approaches may differ substantially, even within the same individual. Furthermore, methodological heterogeneity—including differences in analytical sensitivity, biomarker selection, and study population characteristics—substantially limits comparability across studies. Due to the paucity of human toxicokinetic data, which remains a critical knowledge gap, kinetic parameters such as excretion rates are frequently extrapolated from experimental animal models, most commonly pigs. Although this approach is widely accepted in current risk assessment practice, it represents an additional source of uncertainty that should be explicitly acknowledged when interpreting human exposure estimates [11].

Several studies across Europe have investigated population-level mycotoxins exposure. Exposure levels may vary considerably across regions due to differences in contamination patterns and dietary habits, as demonstrated by recent investigations. In Belgium, fumonisin exposure was not considered to be of major concern; however, exposure to deoxynivalenol (DON), ochratoxin A (OTA), and citrinin (CIT) was identified as potentially problematic. Notably, the DON metabolite deoxynivalenol-15-glucuronide (DON-15-GlcA) was detected in 100% of the analyzed urine samples [12].

In Northern Italy, individuals with celiac disease exhibited higher urinary levels of DON and ZEN, but lower levels of FB_1_, compared with control, highlighting the potential impact of dietary patterns. Although the authors did not consider immediate revision of regulations concerning gluten-free products to be warranted, they emphasized the importance of continuous food monitoring [13]. Similarly, assessments of fumonisin exposure in adults adhering to a gluten-free diet indicated that mycotoxins are widely present in gluten-free products, potentially resulting in chronic dietary exposure [14].

In Southern Italy, the co-occurrence of multiple mycotoxins was observed in all study participants, with urinary biomarkers indicating exposure to DON, FB_1_, OTA, ZEN, α-ZOL, β-ZOL, AFB_1_, and AFM_1_. Biomarker-based exposure estimates were comparable to dietary-based estimates for DON, FB_1_, and ZEN, but higher PDI values were observed for AFB_1_ and OTA [15].

In Swedish adolescents, urinary DON levels were generally low; however, in 2% of individuals the PDI exceeded the tolerable daily intake (TDI). Blood serum analyses indicated a high frequency of OTA contamination in foods consumed by this population. Significant associations were identified between cereal consumption and exposure to DON and OTA. Moreover, OTA exposure was associated with coffee and raisin consumption, while exposure to 2′R-ochratoxin A (2′R-OTA) showed a significant correlation with coffee intake. The authors suggested that 2′R-OTA exposure may be influenced by social and economic factors [16]. The presence of OTA in the human diet, together with current knowledge regarding its toxicity and carcinogenicity, remains a cause for concern [17].

In Germany, mycotoxin exposure levels were generally low, with the exception of DON and DON-glucuronide (DON-GlcA), which exhibited high prevalence; approximately 12% of individuals, the PDI exceeded the TDI. No significant associations were observed between dietary habits and mycotoxin exposure, which may be attributable to the limited sample size and generally low exposure levels [18]. By contrast, a study conducted in the United Kingdom reported a strong correlation between dietary DON intake and urinary DON concentrations [19].

Despite extensive research, no substantial reduction in mycotoxin contamination of cereals and cereal-based products has been observed in recent years. Due to global warming, without effective preventive measures to reduce *Fusarium* and *Aspergillus* contamination in cereals, the frequency and severity of infections are expected to increase in the future. Therefore, continuous monitoring of different consumer populations is recommended [20].

In a previous study, we compared FB_1_ + FB_2_ exposure estimates based on Hungarian consumption and contamination data with exposure estimates derived from urinary FB_1_ + FB_2_ levels in healthy and celiac volunteers. Consistent with Solfrizzo et al. [15], higher PDI values were observed in our study [11].

Given the limited data on how urinary mycotoxin levels correlate with the contamination of consumed foods, the volunteers included in our study kept 3-day dietary records and provided urine samples on the third day.

Therefore, the aim of the present study was to replicate the previously conducted biomarker-based exposure assessment with a larger sample size and to investigate potential associations between urinary biomarkers and food consumption data.

## 2. Results

### 2.1. Urinary Mycotoxin Concentrations

A total of 95 urine samples were collected in Hungary. In 2018, 41 healthy individuals and 19 celiac patients were analyzed, whereas in 2020, 13 healthy individuals and 22 celiac patients were analyzed. Samples were screened for 12 urinary mycotoxins and/or their metabolites using LC-MS/MS. Values above the limit of detection (LOD) were considered positive; however, only toxins detectable above the limit of quantification (LOQ) are reported; Consequently, NIV, AFM_1_, T-2 and HT-2 toxins are not included due to the absence of quantifiable results (Table 1). For FB_2_ in 2020, only one celiac individual exhibited a concentration above the LOQ (2.107 ng/mL); therefore, no mean value is reported in the table.

In 2018, most urine samples from both study groups contained quantifiable levels of ZEN, FB_1_, FB_2_, and OTA. In contrast, in 2020, ZEN remained detectable in almost all samples from healthy individuals, whereas only 50% of celiac samples contained quantifiable levels. DON was quantified in 77% of urine samples from healthy individuals in 2020, but was not detected in celiac patients. De-epoxy-DON (DOM-1) was not detected in 2018, whereas it was present in all samples collected in 2020. The occurrence of FB_1_ was substantially lower in 2020 compared with 2018, and FB_2_ was not detected in 2020. Among all analytes, DON c exhibited the highest mean concentration. OTA was detected in all samples in 2018, but in none of the 2020 samples.

In 2018, the OTA concentration in urine from healthy individuals was significantly higher than in celiac patients (*p* = 0.0028, Wilcoxon test), while in 2020, ZEN concentrations were significantly higher in healthy individuals compared with celiac patients (*p* = 0.0012, Wilcoxon test).

The co-occurrence of multiple mycotoxins is summarized in Table 2. DON and DOM-1, ZEN and its metabolites (α- and β-ZOL), as well as FB_1_ and FB_2_, were grouped together. In 2018, all samples contained at least three co-occurring mycotoxins. In 2020, most samples contained three or more mycotoxins, with a minimum of two toxins detected in each sample.

### 2.2. Exposure Estimation Based on Urine Samples

For the exposure assessment, EDI values were calculated using individual measured parameters including urinary mycotoxin concentrations, daily urine volume, and body weight, combined with 24 h excretion rates determined in pig studies [21] (Table 3 and Table 4). Values below the LOD were replaced with LOD/2, and values below the LOQ were replaced with LOQ/2. For ZEN, the TDI applies to the sum of ZEN and its metabolites. Therefore, following Solfrizzo et al. [15], α-ZOL and β-ZOL concentrations were considered equally weighted when calculating Total ZEN (Total ZEN = ZEN + α-ZOL + β-ZOL). Similarly, the TDI applies to fumonisins B_1_–B_4_, but modified forms (e.g., hydrolyzed) do not need to be included [22]. In this study, we examined the presence of FB_1_ and FB_2_ among the fumonisins. For exposure assessment, the combined FB_1_ + FB_2_ value was used. For DON, the established TDI also covers acetylated forms of DON. However, in this study, only DON and DOM-1 were measured. Since DOM-1 also reflects DON intake, this metabolite was included in the DON-related EDI calculations after accounting for the difference in molar mass (DOM-1 multiplied by 1.057).

Due to marked differences in the occurrence and concentrations of measured mycotoxins between the two study years, EDI values calculated for 2018 and 2020 are presented separately.

In 2018, the highest average EDI values were observed for FB_1_ + FB_2_ and DON in both healthy and celiac groups (Table 3). However, the EDI exceeded the TDI in only 1–2 cases. Exposure to total ZEN remained below the TDI in all individuals. In contrast, OTA exposure markedly exceeded the TDI in both groups with all individuals showing EDI values above the TDI. In the healthy group, the maximum OTA exposure reached approximately 60-fold the TDI.

For the spot urine samples, the sensitivity analysis showed that varying the assumed daily urine volume from 1.5 L to 1.0 or 2.0 L did not affect the proportion of TDI exceedances for total ZEN, OTA, or DON + DOM-1. For FB1 + FB2, the exceedance rate also remained unchanged when 2.0 L/day was assumed, whereas assuming 1.0 L/day reduced the number of participants exceeding the TDI from two (4.9%) to one (2.4%). Overall, the alternative urine-volume assumptions had only a limited effect on TDI exceedance rates and did not alter the overall interpretation of the exposure results.

FB_1_ + FB_2_ exposure was significantly higher in celiac patients than in healthy individuals (*p* = 0.01083, Wilcoxon test).

In 2020 samples, EDI values for DON and its metabolites exceeded the TDI in approximately 31% of cases in the healthy group (Table 4). In the same group, 23% of individuals exceeded the TDI for FB_1_ + FB_2_. Exposure to ZEN and its metabolites, as well as OTA, remained below the TDI in all samples in both groups. In the celiac patients, DON exceeded the TDI in two cases, and FB_1_ + FB_2_ in one case.

No statistically significant differences in EDI values between healthy individuals and celiac patients were observed in 2020 for any of the investigated mycotoxins.

### 2.3. Food Consumption Data

Based on the 3-day dietary records, six food groups relevant for Fusarium toxins were defined, and the mean, minimum, and maximum consumption values were calculated among consumers only. All consumption data were normalized to body weight (Table 5 and Table 6).

In 2020, within the healthy group, only one person consumed products containing mixed gluten-free grains (0.94 g/kg bw), and one consumed muesli mixtures (2.79 g/kg bw). Due to the low number of consumers, these values are not presented in Table 6.

In both sampling years, wheat-based products were consumed in the highest amounts in the healthy group, whereas celiac patients consumed the greatest quantities of products containing mixed gluten-free grains. Notably, some individuals in the celiac group reported limited consuming of wheat-based products (0.19 g/kg bw and 0.23 g/kg bw in 2018 and 2020, respectively), indicating incomplete adherence to a gluten-free diet.

Comparison of the dietary data of healthy and celiac volunteers revealed significant differences between the two groups in most food consumption categories in both years. The only exception in both years was the category of oats and oat-based products. Members of the healthy group consumed lower amounts of corn- and rice-based products. As expected, participants with celiac disease consumed substantially lower amounts of wheat-based products.

### 2.4. Associations Between Urinary Biomarkers and Food Consumption Data

To assess the association between creatinine-adjusted urinary biomarker concentrations and body weight–normalized food consumption data (g/kg bw), Spearman’s rank correlation analyses were performed (Table 7 and Table 8). Only those toxins detected above the limit of detection (LOD) were included.

In 2018, among healthy participants, the consumption of oats and oat-based products showed a significant (FDR-adjusted *p* = 0.001962350574942), moderate positive correlation (r_(s)_ = 0.4970) with urinary FB_2_ levels, and a significant (FDR-adjusted *p* = 0.0222899472), weak positive correlation (r_(s)_ = 0.3753) with OTA concentrations.

Consumption of corn and corn-based products was negatively correlated with urinary alfa-ZOL concentration (r_(s)_ = −0.4564, FDR-adjusted *p* = 0.0126878).

Among celiac patients, a positive trend was observed between the consumption of corn and corn-based products and urinary DON concentrations in celiac patients (r_(s)_ = 0.4464), although statistical significance was not reached (*p* = 0.0554, FDR-adjusted *p* = 0.06745532).

Given the relatively rapid urinary excretion of DON, additional analyses were performed using food consumption data from the day immediately preceding urine collection. No significant correlations were observed in either study group.

In 2020, among healthy participants, the wheat and wheat-based products consumption category showed a significant association only with urinary DON concentration (r_s_ = 0.6868, FDR-adjusted *p* = 0.009509496), showing a moderately strong positive correlation.). The corn and corn-based products consumption category showed a significant and positive correlation with urinary ZEN concentration (r_(s)_ = 0.6137, FDR-adjusted *p* = 0.04134226737).

Within the celiac group, consumption of corn and corn-based products exhibited a positive correlation with DOM-1 concentrations (r_(s)_ = 0.4870, FDR-adjusted *p* = 0.034383779176). Consumption of mixed gluten-free grain products was negatively correlated with urinary DOM-1 (r_(s)_ = −0.4983, FDR-adjusted *p* = 0.045978572031).

## 3. Discussion

### 3.1. Mycotoxin Profiles in Urine Samples

DON is the mycotoxin of the highest prevalence in the Central European region [3,4,5]. In our study, DON was detectable in the urine of 44% of healthy participants and 47% of individuals with celiac disease in 2018, whereas in 2020 it was present in the urine of 77% of healthy participants. It was the mycotoxin detected at the highest concentrations, occurring at one to two orders of magnitude higher levels in urine compared with the other mycotoxins analyzed.

Both in terms of occurrence and measured concentrations, numerous previous European surveys have reported similar findings. In Belgium, DON was detected in the urine of 40% of adults and children (*n* = 394) [12], while in the UK, 100% (*n* = 21) and 94% (*n* = 35) of urine samples from children contained DON [19,26]. High DON prevalence (98%) was also observed in Croatia in the urine of pregnant women (*n* = 40) [27], in 96% of volunteers (*n* = 52) in Southern Italy [15], in 63% of participants in Portugal (*n* = 94) [28], and in 100% of total DON (sum of DON, DON-GlcA, and DOM glucuronide) in Norway (*n* = 257) [29]. A relatively low incidence of 4.8% was reported in adolescents in Sweden [16]. Most of the studies cited focused exclusively on DON and did not report the presence of its metabolites, such as DON-15-GlcA, DON-3-glucuronide, or DOM-1.

DOM-1 is a gut microbiota-derived metabolite of DON. In humans, intestinal deepoxidase activity appears to be limited, suggesting a low capacity for microbial detoxification of DON [30]. DOM-1 formed in the intestine is absorbed and excreted mainly as DOM-GlcA (glucuronide) in urine [31]. Consistent with the generally low deepoxidation activity in humans, Turner et al. [32] reported that DOM-1 equivalents represent only ~1% of total DON equivalents. In a French cohort, DOM-1 was detected in 26 of 76 urine samples (mean: 0.2 ng/mL), exclusively among cattle farm workers; the authors suggested that exposure to bovine rumen microbiota may have contributed to enhanced deepoxidase activity [32]. Even in these cases, DOM-1 accounted for less than 5% of total DON.

Similarly, Heyndrickx et al. [12] detected DOM-Glc in 17% of children and 22% of adults (*n* = 394), while free DOM-1 was not evaluated due to the lack of β-glucuronidase pretreatment. These findings collectively indicate that DOM-1 formation in humans is generally limited and variable.

In our study series, no DOM-1 was detected in samples collected in 2018, whereas measurable concentrations were present in all samples in 2020. The reasons for this difference may be related to temporal variability in exposure patterns, interindividual metabolic differences, or other factors not assessed in the present study. In healthy individuals, DOM-1 was detected alongside DON and represented approximately 30% of DON concentrations on average, indicating partial microbial transformation. In contrast, in patients with coeliac disease, DOM-1 was detected in the absence of measurable DON (all DON values < LOD), which may reflect altered DON metabolism, differences in gut microbiota composition, or distinct dietary exposure patterns in this group.

ZEN was the second-most prevalent mycotoxin in our region [3,4,5]. In 2018, it was detected in all samples except one, indicating widespread exposure. A similar pattern was observed in 2020 in the healthy group; however, only half of the coeliac patients had urinary ZEN concentrations above the LOQ, suggesting possible differences in dietary exposure or metabolism between the groups.

Cirlini et al. [13] reported significantly higher urinary DON and ZEN concentrations in coeliac patients compared to healthy individuals, which they attributed to the potential contamination of gluten-free cereal-based products. In contrast, in our 2018 cohort, no significant differences were found between coeliac and healthy participants in DON or ZEN excretion. Interestingly, in 2020, urinary ZEN concentrations were significantly higher in the healthy group (*p* = 0.0012), and DON levels showed a similar trend, although this difference did not reach statistical significance. These findings suggest temporal variability and indicate that exposure patterns in our population may differ from those previously reported, possibly reflecting regional dietary habits or changes in food sourcing.

Fumonisins rank as the third-most prevalent mycotoxin group in the Central European region [3,4,5]. In 2018, both FB_1_ and FB_2_ were detected in 100% of samples in both groups, indicating ubiquitous exposure. However, in 2020, FB_1_ was detected in only 31% of healthy participants and 59% of coeliac patients, while FB_2_ was not detected except in a single sample from the coeliac group.

Although Cirlini et al. [13] observed lower urinary FB_1_ concentrations in coeliac patients compared to healthy controls, we did not identify significant differences between the two groups in either study year. The marked year-to-year variation observed in our dataset further supports the influence of changing dietary sources or market availability of cereal-based products on fumonisin exposure.

According to the WHO [2], the mycotoxins posing the greatest risk to human health, alongside *Fusarium* toxins, are aflatoxins and ochratoxin A (OTA). However, their prevalence in our region is relatively low, around 10% [3,4,5]. Consistently, AFB_1_ was not detected in any of our urine samples, in either study group or year. In contrast, OTA was measurable above the LOQ in all 2018 samples. By 2020, all samples fell below the LOQ, although concentrations above the LOD were still observed in approximately 85% of healthy participants and 59% of coeliac patients. OTA was also measurable in southern Italy [33].

These findings align with those of Heyndrickx et al. [12], who highlighted concerning urinary OTA levels in Belgian participants, and with the 2020 results of Lemming et al. [16], who reported frequent OTA contamination in foods consumed by Swedish adolescents. In our study, urinary OTA concentrations were significantly higher in healthy participants in 2018 (*p* = 0.0028). This difference was not observed in 2020, likely due to all measurements falling below the LOQ.

The co-occurrence of multiple mycotoxins in human urine has been reported previously. Solfrizzo et al. [15] detected DON, FB_1_, OTA, ZEN, α-ZOL, β-ZOL, and AFB_1_ simultaneously in urine samples from volunteers in Southern Italy. In line with these findings, we observed a similarly high prevalence and frequent co-occurrence of these toxins in both study years.

### 3.2. Biomarker-Based Exposure Assessment

In the study of Solfrizzo et al. [15], urinary biomarker-based PDIs for DON were comparable to dietary intake-based estimates reported in the literature. Similarly, Lemming et al. [16] found that only 2% of Swedish adolescents exceeded the TDI based on biomarker assessments. In our 2018 study, a similar pattern was observed: 4.9% of healthy participants and 5.3% of coeliac patients exceeded the DON TDI. By contrast, in 2020, the proportion exceeding the TDI increased markedly to 30.8% in healthy participants, while remaining 9.1% in the coeliac group.

Gerding et al. [18], in their assessment of the German population, reported intermediate results, with 12% of samples exceeding the DON TDI.

Heyndrickx et al. [12] also highlighted concerning urinary DON levels in Belgium. Depending on the methodology applied—such as whether differences in molecular weight of DON metabolites were considered or whether morning urine was used to estimate total daily excretion—16–69% of the population was estimated to exceed the tolerable intake.

Overall, these findings underscore both regional and temporal variability in DON exposure and highlight the value of urinary biomarkers for more accurate exposure assessment.

In the 2014 study by Solfrizzo et al. [15], urinary biomarker-based PDI values for ZEN were comparable to dietary intake-based PDI reported in the literature, remaining well below the TDI. Although ZEN shows high prevalence in our region [3,4,5] and was frequently detected in the samples we analyzed, all measured concentrations remained below the TDI in both study years.

Similarly, in the case of FB_1_, Solfrizzo et al. [15] reported urinary biomarker-based PDI values comparable to dietary intake-based PDI. Zentai et al. [8], using Hungarian food consumption and mycotoxin data, estimated that the combined FB_1_ + FB_2_ EDI exceeded the TDI in only 1% of cases. Our own urinary biomarker-based assessment paints a somewhat less favorable picture: in 2018, 4.9% of healthy participants and 5.3% of coeliac patients exceeded the TDI. The relatively small difference between the two groups was found statistically significant (*p* = 0.01, Wilcoxon test). In 2020, 23.1% of healthy participants and 4.6% of coeliac patients exceeded the TDI. Interestingly, despite the seemingly large difference between the groups, the Wilcoxon test did not find it statistically significant (*p* = 0.946), likely due to the presence of outlier values and high variability within the study population.

Solfrizzo et al. [15] reported that urinary biomarker-based PDI values for OTA substantially exceeded dietary intake-based estimates. Similarly, Lemming et al. [16] found that foods consumed by Swedish adolescents were frequently contaminated with OTA. Consistent with these findings, all urine samples in our 2018 study exceeded the OTA TDI. In contrast, in 2020, none of the samples exceeded the TDI, highlighting temporal variability in exposure.

For AFB_1_, Solfrizzo et al. [15] also reported urinary PDI values well above dietary intake estimates. In our study, however, AFB_1_ was undetectable in all samples across both years, likely reflecting climatic differences between Central and Southern Europe.

### 3.3. Associations Between Dietary Intake and Biomarker Levels

Lemming et al. [16] reported an association between cereal consumption and DON exposure in Swedish adolescents. In our study, cereals were grouped into specific categories, and no significant correlations between wheat and wheat product consumption and DON exposure were observed in 2018. However, in 2020, a moderately strong and significant positive correlation was found between wheat and wheat product consumption and DON exposure in the healthy group (r(s) = 0.6868, FDR-adjusted *p* = 0.009509496).

ZEN has frequently been detected in maize [34]. In 2020, a strong positive correlation was observed between maize and maize product consumption and ZEN exposure in the healthy group (r(s) = 0.6137, FDR-adjusted *p* = 0.04134226737).

Fumonisins are frequently found in maize and maize products but can also occur in other cereals, including wheat, rice, barley, and oats [34]. In our study, no significant correlations were observed between the examined fumonisins (FB1 and FB2) and maize or maize product consumption. However, a significant positive correlation was observed between oat and oat product consumption and FB2 exposure in the healthy group in 2018 (r(s) = 0.4970, FDR-adjusted *p* = 0.001962350574942).

Although these correlations are consistent with biologically plausible associations between specific cereal groups and mycotoxin exposure, they do not establish the respective foods as sources of exposure, since mycotoxin concentrations were not measured in the foods actually consumed by the participants. Therefore, the observed correlations should be interpreted as associations between dietary patterns and urinary biomarker levels rather than as evidence of specific dietary sources of mycotoxin exposure.

An important limitation of the present exposure assessment is the uncertainty associated with the urinary excretion fractions used for the back-calculation of EDI values. For several mycotoxins, sufficiently robust human excretion data are not available; therefore, excretion fractions derived from pig studies were used as the best available estimates. Since the urinary excretion fraction appears in the denominator of the EDI equation, EDI is inversely proportional to this parameter, and uncertainty in the excretion fraction translates directly into uncertainty in the estimated intake. Therefore, EDI values calculated using animal-derived excretion fractions should be interpreted as estimates subject to uncertainty, and more robust human toxicokinetic data are needed to improve the accuracy of biomonitoring-based exposure assessments.

Marked differences in the occurrence of OTA and DOM-1 were observed between the participant groups sampled in 2018 and 2020. However, these differences should not be interpreted solely as interannual variability, as the two surveys involved different participants with potentially different dietary habits and substantially different sample sizes. In addition, exposure to OTA may originate from several dietary sources, including foods and beverages not specifically evaluated in the present correlation analysis, whereas DOM-1 is a metabolite of DON. Consequently, several factors may have contributed to the observed differences in OTA and DOM-1 occurrence. The present study design does not allow the relative contribution of these factors to be determined, and any explanation for the observed differences would therefore be speculative.

## 4. Conclusions

In conclusion, our results generally corroborate findings reported in the literature, although some differences were observed. Notably, the results from the two years of our study often differ from each other, highlighting the temporal variability of mycotoxins’ occurrence and the influence of environmental and dietary factors, such as cereal consumption patterns on human exposure.

Our findings further emphasize the value of urinary biomarkers for accurate exposure assessment and demonstrate that specific cereal types and dietary patterns influence mycotoxin exposure. Moreover, substitutions within gluten-free diets could potentially modulate biomarker levels.

## 5. Materials and Methods

### 5.1. Study Population, Dietary Assessment and Urine Sample Collection

The study was conducted in accordance with the Declaration of Helsinki. The study protocol was approved by the Scientific and Research Ethics Committee of the Medical Research Council (Hungary) at its meeting on 29 August 2017 (ID 32958-3/2017/EKU). The approval was valid for four years from the date of issuance, thereby covering bought sampling years. Written informed consent was obtained from all volunteers included in the study.

To assess human exposure to DON, FB_1_, ZEN, and OTA, 60 individuals (41 healthy and 19 coeliac patients) were recruited in 2018 from Somogy, Baranya, and Pest counties (Hungary), and 35 individuals (13 healthy and 22 coeliac patients) in 2020. For participants with coeliac disease, we expected different exposure patterns associated with adherence to a gluten-free diet, which could lead to either lower or higher overall exposure. Prior to the start of the study, participants were required to answer general questions regarding their health status. Individuals diagnosed with and/or presenting symptoms of liver or kidney disease, or any chronic disease, were excluded from the study due to the potential interference with mycotoxin and creatinine metabolism. The sex (male/female), age, height, and body weight of the volunteers were recorded.

Each volunteer recorded all foods consumed on three non-consecutive days in a food diary. Of the three days, two were weekdays and one was a weekend day. Urine collection was performed on the fourth day following the third recorded day (with a maximum of 7 days between the first recorded day and urine collection).

For each meal, participants were asked to provide the name of the food (brand name and specific quality characteristics), the name of the prepared dish and its method of preparation (boiling, steaming, deep-frying, breading, thickening, etc.), and portion size (based on a separate guideline). They were also asked to specify precisely the quantity and quality characteristics of fluids consumed. Before the start of the study, participants received a consultation and detailed written instructions on how to complete the diary. Upon submission, the diaries were validated by trained staff members. Food consumption was recorded, and dietary intake data were calculated using the Hungarian NutriComp 5.0 software (NutriComp Ltd., Budapest, Hungary).

Each participant collected a 24 h urine sample according to the specified protocol in 2.5 L plastic urine collection containers. Collection began with the first morning void and ended at the same time the following day. Where this was not feasible (*n* = 12), a spot urine sample was collected, and exposure estimation was calculated using an average daily urine volume of 1.5 L [19].

The total volume of the 24 h urine samples was measured with milliliter accuracy. After thorough mixing, two 100 mL aliquots were taken for creatinine and biomarker analyses. Samples were stored at −20 °C.

### 5.2. Urinary Multi-Mycotoxin Biomarker Analysis by LC–MS/MS

Concentrations of ZEN, DON, FB_1_, FB_2_, OTA, T-2 toxin, HT-2 toxin, and nivalenol (NIV), as well as their metabolites (deepoxy-deoxynivalenol, α-zearalenol, β-zearalenol, aflatoxin M_1_), were analyzed in urine samples at the Institute of Sciences of Food Production (ISPA-CNR), Italy.

Urinary biomarker analyses were performed as previously described by Szabó-Fodor et al. [11] with minor modifications. Briefly, urine samples were thawed and centrifuged (4500 rpm, 10 min). An aliquot of 6 mL urine was incubated overnight at 37 °C with 300 µL β-glucuronidase/sulfatase to hydrolyze glucuronide and sulfate conjugates of mycotoxins and/or their metabolites into their free forms. After incubation, samples were diluted with 6 mL Milli-Q water, vortex-mixed and centrifuged again (4500 rpm, 10 min). Sample clean-up was performed using Myco6in1+™ multi-antibody immunoaffinity column (Vicam, Watertown, MA, USA) connected in tandem with an OASIS^®^ HLB column (Waters, Milford, MA, USA). The Oasis HLB cartridge was conditioned with 2 mL methanol followed by 2 mL water. After loading the entire sample, the two cartridges were separated and washed individually. The Oasis HLB cartridge was washed with 1 mL methanol/water (20:80, *v*/*v*), while the Mycoto6in1+ column was washed with 4 mL Milli-Q water. Analytes were eluted from the Oasis HLB cartridge with 1 mL methanol/water (40:60, *v*/*v*), whereas the Myco6in1+ column was eluted with 3 mL methanol followed by 2 mL water. The two purified eluates were combined, evaporated to dryness under a gentle air stream at 50 °C, and reconstituted in 200 µL of mobile phase consisting of methanol/water (20:80, *v*/*v*) containing 0.5% acetic acid, filtered with a regenerated cellulose filter (0.20 µm) syringe and analyzed by LC–MS/MS.

Quantification of mycotoxins was performed using matrix-matched calibration curve prepared using blank urine obtained by pooling samples from three individuals who had followed a grain-free diet for three days. The pooled urine was analyzed without the addition of mycotoxin standards to assess background levels of target analytes, which ranged from not detected to below the LOQ. Standard solutions of ZEN, DON, FB_1_, FB_2_, OTA, T-2, HT-2, NIV, DOM-1, α-ZOL, β-ZOL and AFM_1_ were used to prepare mixed spiking solutions. Appropriate amounts of these solutions were added to the purified extracts to prepare six matrix-matched calibration levels at increasing concentrations. The performance of each analytical sequence was monitored by periodically injecting control standard solutions at known concentrations to verify the stability of the instrumental response and the reliability of the quantitative analysis. Each matrix-matched calibration level was analyzed in triplicate to assess the repeatability of the analytical response. The analytical procedure was previously validated by Solfrizzo et al. [35], with mean recoveries ranging from 62% to 96% and relative standard deviations (RSDs) ranging from 3% to 20%. Additional recovery experiments were performed for NIV, T-2 and HT-2, resulting in mean recoveries of 82%, 79% and 102%, respectively. For each analyte, the LOD and LOQ were determined as the concentrations corresponding to signal-to-noise ratios (S/N) of 3 and 10, respectively. Samples collected in 2018 and 2020 were analyzed using two different LC–MS/MS systems. The 2018 samples were analyzed using a Q Sight LX50 system, whereas the 2020 samples were analyzed using an API 5000 system. Consequently, LOD and LOQ values were determined separately for each data set and reflect the different analytical performance of the two LC-MS/MS systems (Table 9).

Urinary creatinine concentrations were determined using a Roche/Hitachi cobas c 501/502 analyzer (Roche Diagnostics GmbH, Mannheim, Germany) with the Creatinine Jaffé Gen.2 700 assay, according to the manufacturer’s instructions.

### 5.3. LC-MS/MS Equipment and Parameters

A triple quadrupole API 5000 system (Applied Biosystems, Foster City, CA, USA), equipped with an ESI interface and an Acquity UPLC system comprising a binary pump and a micro autosampler from Waters (Milford, MA, USA), was used for LC-MS/MS analyses of 2020 samples. The LC-MS/MS conditions for the analytes included in the original method were those described by Solfrizzo et al. [15]. For separation of mycotoxins, an Acquity UPLC BEH phenyl column (2.1 mm × 150 mm, 1.7 μm particles; Waters) was used, and the column oven was set at 40 °C. The flow rate of the mobile phase was 250 μL/min, and the injection volume was 10 μL. For multi-biomarker separation a multiple linear binary linear gradient of acidic MeOH (containing 0.5% acetic acid) in water (containing 0.5% acetic acid) was developed and used as mobile phase as follows: from 20% to 80% MeOH in 5 min, then maintained at 80% MeOH for 5 min, then brought to 20% MeOH in 0.5 min and left to equilibrate for 4.5 min before the next run. For LC-MS/MS analyses, the ESI interface was used in positive ion mode for AFM1, FB1, T-2, HT-2 and OTA and in negative ion mode for NIV, DON, DOM-1, ZEA, α-ZOL and β-ZOL. The mass spectrometer operated in MRM (multiple reaction monitoring) mode and the optimized MS/MS conditions for each analyte are listed in Solfrizzo et al., [15]. Since NIV, T-2 and HT-2 toxins were not included in that method, the corresponding MRM transitions and compound-dependent parameters were optimized separately and are reported in Table 10.

### 5.4. Urinary Biomarker-Based Exposure Calculation

Although previous studies report PDI values, in the present work we use the term Estimated Daily Intake (EDI) for consistency with international terminology. The individual EDI of each mycotoxin was calculated using the following equation [15]:

$$\mathrm{EDI}=C\times \frac{V}{W}\times \frac{100}{E}$$

where

EDI: Estimated daily intake of the mycotoxin (µg/kg bw/day).

C: Biomarker concentration in urine (µg/L).

V: 24 h urine volume (L).

W: Body weight (kg).

E: Mean urinary excretion rate in pigs according to Gambacorta et al. [21], i.e., 36.8% for ZEN, 27.9% for DON, 2.6% each for FB_1_ and OTA.

For 12 participants, spot urine samples rather than 24 h urine samples were available; therefore, a daily urine volume of 1.5 L was assumed for the calculation of EDI. All 12 participants belonged to the 2018 healthy group (12/41, 29.3%), whereas all participants in the other study groups provided 24 h urine samples. To evaluate the potential effect of the assumed urine volume, a sensitivity analysis was performed by recalculating the EDI values of these 12 participants using alternative daily urine volumes of 1.0 and 2.0 L.

The calculated EDI values were analyzed using R software, version 4.4.1, to assess whether statistically significant differences existed between the two groups (healthy vs. coeliac). Normality was tested with the Shapiro–Wilk test, and depending on the outcome, either a *t*-test or a Wilcoxon test was applied.

The present study focuses on exposure assessment based on urinary biomonitoring data; a full risk characterization was beyond the scope of this work.

### 5.5. Associations Between Food Intake and Urinary Mycotoxin Concentrations

The dietary intake data were normalized to body weight (kg), and the average consumption over the three recorded days was used for further calculations. Urinary mycotoxin concentrations were corrected for creatinine content by dividing the mycotoxin concentration by the urinary creatinine concentration and expressed as nanograms of mycotoxin per milligram of creatinine (ng/mg creatinine). For values below the limit of detection (LOD), LOD/2 was used, and for values below the limit of quantification (LOQ), LOQ/2 was applied in subsequent calculations, following the middle-bound substitution approach previously applied in mycotoxin biomonitoring studies [11,16,36,37]. To limit the potential influence of this substitution approach on the correlation analyses, mycotoxins with quantifiable concentrations in less than 20% of the samples were excluded from these analyses.

Consumed foods were grouped into six categories considered relevant sources of Fusarium mycotoxins according to the literature, and statistical analyses were performed for these groups:

Wheat and wheat products;

Maize and maize products;

Rice and rice products;

Oats and oat products;

Gluten-free foods containing mixed cereals;

Muesli mixes.

Statistical analyses were performed using R software, version 4.4.1. Spearman correlation tests were used to evaluate the associations between urinary mycotoxin concentrations and the consumption of different food groups (r_(s)_), along with significance levels (*p*). The Benjamini–Hochberg false discovery rate (FDR) method was applied to adjust the *p*-values for multiple testing in the correlation analyses, with adjusted *p*-values < 0.05 considered statistically significant.

Statistical limitations: To reduce the risk of unreliable correlations due to sparse data, food groups consumed by only 2 or 3 individuals were not included in the correlation analysis. Mycotoxins with quantifiable concentrations in less than 20% of the samples were also excluded, as the large proportion of values below the LOQ precluded a reliable assessment of correlations.

The relatively small sample sizes of the individual subgroups limited the statistical analyses that could be reliably performed. In particular, the small subgroup sizes, together with the heterogeneity in mycotoxin occurrence and dietary patterns between the sampling years and study groups, limited the suitability and interpretability of a multivariable analysis. Combining the subgroups into a single multivariable model was not considered appropriate, as this could obscure group- and year-specific associations. Therefore, subgroup-specific Spearman correlation analyses were retained, and the observed associations should be interpreted with caution given the limited sample sizes.

The authors declare that artificial intelligence (AI) tools were used solely for language editing and, in some cases, translation purposes. No scientific content, data interpretation, or study design was generated or influenced by AI.

## Figures and Tables

**Table 1:** Mycotoxin concentrations (ng/mL) measured in the urine samples of healthy people and celiac patients.

Mycotoxin	*n* Above LOQ	Mean	Median	SD	Min	Max	*n* Above LOQ	Mean	Median	SD	Min	Max
	2018						2020					
Healthy people (ng/mL)												
ZEN	40	0.043	0.029	0.036	0.012	0.174	12	0.178 *	0.051	0.403	0.020	1.449
DON	18	5.163	3.824	4.167	2.010	18.947	10	8.561	7.474	5.785	1.421	22.429
DOM-1	0	-	-	-	-	-	13	2.721	1.965	1.684	0.930	6.404
FB_1_	41	0.358	0.247	0.385	0.166	2.285	4	1.116	1.095	0.925	0.009	2.266
FB_2_	41	0.060	0.032	0.134	0.008	0.875	0	-	-	-	-	-
OTA	41	0.166 *	0.124	0.155	0.071	1.024	0	-	-	-	-	-
α-ZOL	34	0.017	0.015	0.012	0.004	0.063	12	0.050	0.044	0.014	0.035	0.071
β-ZOL	8	0.017	0.013	0.008	0.013	0.033	3	0.032	0.026	0.015	0.022	0.049
Celiac people (ng/mL)												
ZEN	19	0.041	0.034	0.030	0.011	0.121	11	0.016 *	0.002	0.019	0.002	0.060
DON	9	5.59	5.071	1.861	3.876	9.484	-	-	-	-	-	-
DOM-1	-	-	-	-	-	-	22	4.332	2.649	6.005	0.768	25.067
FB_1_	19	0.301	0.285	0.085	0.173	0.495	13	1.067	0.009	3.503	0.009	12.705
FB_2_	19	0.046	0.029	0.046	0.016	0.226	-	-	-	-	-	-
OTA	19	0.089 *	0.078	0.044	0.052	0.243	-	-	-	-	-	-
α-ZOL	11	0.022	0.016	0.019	0.004	0.071	18	0.041	0.041	0.005	0.034	0.055
β-ZOL	-	-	-	-	-	-	7	0.017	0.013	0.006	0.013	0.025

* Significant difference in OTA concentration (*p* = 0.0028, Wilcoxon test) in 2018 and ZEN (*p* = 0.0012, Wilcoxon test) in 2020 between healthy and celiac people. Abbreviations: LOQ, limit of quantification; SD, standard deviation; ZEN, zearalenone; DON, deoxynivalenol; DOM-1, de-epoxy-deoxynivalenol; FB_1_, fumonisin B_1_; FB_2_, fumonisin B_2_; OTA, ochratoxin A; α-ZOL, alpha-zearalenol; β-ZOL, beta-zearalenol.

**Table 2:** The co-existence of mycotoxins in human urine *.

Number of Co-Occurring Mycotoxins **	Number of Samples Showing Co-Occurrence		Percentage of Samples Showing Co-Occurrence (%)	
	2018	2020	2018	2020
Healthy people (ng/mL)				
2	-	1	-	7.7
3	23	6	56.1	46.1
4	17	4	41.5	30.8
5	1	2	2.4	15.4
Celiac people (ng/mL)				
2	-	3	-	13.6
3	10	7	52.6	31.8
4	9	8	47.4	36.4
5	-	3	-	13.6

* Values above the limit of detection (LOD) were considered positive. ** For the purpose of assessing co-occurrence, parent mycotoxins and their metabolites were counted as a single mycotoxin. Thus, DON and DOM-1, as well as ZEN and its metabolites, were considered as single mycotoxins. Similarly, FB_1_ and FB_2_ were counted as one mycotoxin group in the table.

**Table 3:** Urinary mycotoxin concentrations and calculated EDI in healthy and celiac individuals in Hungary, 2018.

		Concentration (µg/L)		Excretion Rate in Pig (%) *	EDI (µg/kg bw/day)		TDI ** (µg/kg bw/day)	EDI/TDI %		*n* Above TDI	% Above TDI
		Mean	Max		Mean	Max		Mean	Max		
Healthy people *n* = 41	Total ZEN	0.062	0.199	36.8	0.004	0.020	0.25	1.64	8.13	0	0
	DON + DOM-1	2.376	19.021	27.9	0.202	1.623	1.00	20.17	162.32	2	4.9
	FB_1_ + FB_2_	0.327	1.698	2.6	0.280 ^b^	1.183	1.00	28.03	118.32	2	4.9
	OTA	0.166	1.024	2.6	0.144	1.055	0.017	848.38	6203.63	41	100.0
Celiac patients *n* = 19	Total ZEN	0.059	0.152	36.8	0.006	0.023	0.25	2.25	9.28	0	0
	DON + DOM-1	2.753	9.556	27.9	0.336	1.976	1.00	33.59	197.61	1	5.3
	FB_1_ + FB_2_	0.275	0.445	2.6	0.374 ^a^	1.026	1.00	37.37	102.65	1	5.3
	OTA	0.089	0.243	2.6	0.126	0.560	0.017	739.23	3296.97	19	100.0

* According to Gambacorta et al. [21]. ** TDI values for mycotoxins: ZEN 0.250 µg/kg bw/day [23], FB_1_ 1.0 µg/kg bw/day [22], DON 1.0 µg/kg bw/day [24], OTA 0.017 µg/kg bw/day (derived from 0.120 µg/kg bw/week, [25]). Total ZEN = ZEN + (α-ZOL) + (β-ZOL). DOM-1 was taken into account with a multiplier of 1.057 to compensate for the difference in molar mass compared to DON. ^a,b^ statistical difference between healthy and celiac people in FB_1_ + FB_2_ exposure (*p* = 0.01083, Wilcoxon test). Abbreviations: EDI, estimated daily intake; TDI, tolerable daily intake; ZEN, zearalenone; DON, deoxynivalenol; DOM-1, de-epoxy-deoxynivalenol; FB_1_, fumonisin B_1_; FB_2_, fumonisin B_2_; OTA, ochratoxin A; α-ZOL, alpha-zearalenol; β-ZOL, beta-zearalenol.

**Table 4:** Urinary mycotoxin concentrations and calculated EDI in healthy and celiac individuals in Hungary, 2020.

		Concentration µg/L *		Excretion Rate in Pig (%) *	EDI (µg/kg bw/day)		TDI ** (µg/kg bw/day)	EDI/TDI %		*n* Above TDI	% Above TDI
		Mean	Max		Mean	Max		Mean	Max		
Healthy people *n* = 13	Total ZEN	0.221	1.508	36.8	0.018	0.147	0.25	7.24	58.78	0	0
	DON + DOM-1	9.477	26.581	27.9	0.783	1.774	1.00	78.34	177.43	4	30.8
	FB_1_ + FB_2_	0.348	2.268	2.6	0.310	1.745	1.00	31.01	174.48	3	23.1
	OTA	0.0004	0.0005	2.6	0.0004	0.0009	0.017	2.48	5.03	0	0
Celiac patients *n* = 22	Total ZEN	0.050	0.114	36.8	0.004	0.012	0.25	1.76	4.96	0	0
	DON + DOM-1	4.645	26.563	27.9	0.638	5.479	1.00	63.82	547.88	2	9.1
	FB_1_ + FB_2_	0.736	14.812	2.6	0.620	11.832	1.00	61.97	1183.22	1	4.5
	OTA	0.0004	0.0005	2.6	0.0004	0.001	0.017	2.57	6.51	0	0

* According to Gambacorta et al. [21]. ** TDI values for mycotoxins: ZEN 0.250 µg/kg bw/day [23], FB_1_ 1.0 µg/kg bw/day [22], DON 1.0 µg/kg bw/day [24], OTA 0.0017 µg/kg bw/day (derived from 0.120 µg/kg bw/week, [25]. Total ZEN= ZEN + (α-ZOL) + (β-ZOL). DOM-1 was taken into account with a multiplier of 1.057 to compensate for the difference in molar mass compared to DON. Abbreviations: EDI, estimated daily intake; TDI, tolerable daily intake; ZEN, zearalenone; DON, deoxynivalenol; DOM-1, de-epoxy-deoxynivalenol; FB_1_, fumonisin B_1_; FB_2_, fumonisin B_2_; OTA, ochratoxin A; α-ZOL, alpha-zearalenol; β-ZOL, beta-zearalenol.

**Table 5:** Grain consumption normalized to body weight in healthy (*n* = 41) and celiac (*n* = 19) individuals in 2018.

Food Group	*n* *	Mean (g/kg bw)	SD	Min (g/kg bw)	Max (g/kg bw)
Healthy people (*n* = 41)					
Wheat and its products	38	2.24 ^(1)^	1.10	0.56	4.45
Mixture of g.f. grains **	2	0.15 ^(2)^	0.06	0.11	0.19
Corn and its products	12	0.38 ^(3)^	0.41	0.05	1.47
Rice and its products	20	0.37 ^(4)^	0.35	0.05	1.69
Oat and its products	14	0.32	0.37	0.05	1.50
Coeliac people (*n* = 19)					
Wheat and its products	8	0.19 ^(1)^	0.28	0.02	0.83
Mixture of g.f. grains **	18	3.01 ^(2)^	1.43	0.10	5.29
Corn and its products	16	1.28 ^(3)^	1.20	0.05	4.95
Mixture of muesli	4	0.56	0.30	0.16	0.90
Rice and its products	16	0.77 ^(4)^	0.65	0.17	2.90
Oat and its products	6	0.55	0.25	0.30	1.00

* Number of people who consume that certain food type. ** Mixture of gluten-free grains. Significant differences between healthy and celiac people are marked with numbers. Levels of significance (Wilcoxon test) *p* = 0.000254 ^(1)^, 0.0002141 ^(2)^, 0.002861 ^(3)^, 0.0009208 ^(4)^.

**Table 6:** Grain consumption normalized to body weight in healthy (*n* = 13) and celiac (*n* = 22) individuals in 2020.

Food Group	*n* *	Mean (g/kg bw)	SD	Min (g/kg bw)	Max (g/kg bw)
Healthy people (*n* = 13)					
Wheat and its products	13	7.21 ^(1)^	3.86	3.28	14.96
Corn and its products	9	0.92 ^(2)^	0.35	0.38	1.30
Rice and its products	3	0.80 ^(3)^	0.80	0.20	1.70
Oat and its products	2	1.01	0.18	0.88	1.13
Coeliac people (*n* = 22)					
Wheat and its products	13	0.23 ^(1)^	0.37	0.03	1.42
Mixture of g.f. grains **	18	3.18	2.77	0.13	8.50
Corn and its products	18	2.1 ^(2)^	1.17	0.41	4.35
Mixture of muesli	4	0.70	0.31	0.43	0.98
Rice and its products	20	1.61 ^(3)^	1.23	0.13	4.50
Oat and its products	5	0.93	1.08	0.15	2.79

* Number of people who consume that certain food type. ** Mixture of gluten-free grains. Significant differences between healthy and celiac people are marked with numbers. Levels of significance (Wilcoxon test) *p* = 0.0002441 ^(1)^, 0.003264 ^(3)^, (*t*-test) *p* = 0.01788 ^(2)^.

**Table 7:** Correlations Between Food Consumption and Urinary Toxin Levels in Healthy and Celiac Individuals in 2018.

r_(s)_ *	*n*	DON	FB_1_	FB_2_	OTA	ZEN	α.ZOL
Healthy people							
Wheat and its products	38	0.1282	−0.1960	−0.0699	−0.1157	−0.2584	−0.2826
Corn and its products	12	0.0178	0.1125	0.1207	0.1441	−0.2947	**−0.4564 ^(1)^**
Rice and its products	20	0.0704	−0.0597	−0.1150	0.0197	0.0949	0.0075
Oat and its products	14	−0.0478	0.2781	**0.4970 ^(2)^**	**0.3753 ^(3)^**	0.1266	0.1286
Celiac people							
Wheat and its products	8	0.0801	−0.0020	0.2539	0.3047	−0.1601	0.0332
Mixture of gluten-free grains	18	0.3807	0.0719	0.0386	0.2965	0.0193	−0.0596
Corn and its products	16	0.4464	0.0791	0.0369	0.1371	0.2601	0.1986
Mixture of muesli	4	0.0566	0.1476	0.0639	0.0885	0.1353	−0.0344
Rice and its products	16	−0.2654	0.0246	0.1142	−0.0176	0.0967	−0.0053
Oat and its products	6	0.0191	−0.2764	−0.0532	−0.3764	−0.0893	−0.0468

* r_(s)_: Spearman’s rank correlation coefficient. Significant correlations are marked in bold. Levels of significance (*p*=): ^(1)^ 0.0027, ^(2)^ 0.0009, ^(3)^ 0.0156. *p*-values adjusted using Benjamini–Hochberg method (*p*=): ^(1)^ 0.0126878, ^(2)^ 0.001962350574942, ^(3)^ 0.0222899472.

**Table 8:** Correlations Between Food Consumption and Urinary Toxin Levels in Healthy and Celiac Individuals in 2020.

r_(s)_ *	*n*	DOM-1	DON	FB_1_	NIV	OTA	ZEN	α-ZOL	β-ZOL
Healthy people									
Wheat and its products	13	0.1703	**0.6868 ^(1)^**	−0.2308	nr	−0.1319	0.2473	0.0769	−0.4396
Corn and its products	9	−0.0530	−0.3180	0.0112	nr	0.0865	**0.6137 ^(2)^**	0.2120	0.1590
Celiac people									
Wheat and its products	13	0.1246	nr	0.0287	−0.0135	nr	0.2848	0.2029	−0.0544
Mixture of gluten-free grains	**18**	**−0.4983 ^(3)^**	nr	−0.3511	−0.3703	nr	−0.3749	−0.3375	−0.1065
Corn and its products	**18**	**0.4870 ^(4)^**	nr	0.3613	0.3137	nr	0.1863	0.2033	−0.0561
Mixture of muesli	4	0.1603	nr	−0.0470	−0.0319	nr	0.2333	0.1116	−0.0378
Rice and its products	20	−0.1480	nr	−0.1135	0.0090	nr	−0.0870	−0.1966	0.1751
Oat and its products	5	0.1392	nr	0.1576	0.3468	nr	−0.2407	0.0730	0.1038

* r(s): Spearman’s rank correlation coefficient. Significant correlations are marked in bold. Levels of significance (*p*=): ^(1)^ 0.0095, ^(2)^ 0.0257, ^(3)^ 0.0183, ^(4)^ 0.0215. *p*-values adjusted using Benjamini–Hochberg method (*p*=): ^(1)^ 0.009509496, ^(2)^ 0.04134226737, ^(3)^ 0.045978572031, ^(4)^ 0.034383779176. nr: not relevant (high proportion of <LOQ).

**Table 9:** Limits of detection (LOD) and quantification (LOQ) for urinary mycotoxins and their metabolites determined by LC–MS/MS (ng/mL).

Mycotoxin	LOD (ng/mL)		LOQ (ng/mL)	
	QSight LX50 LC-MS/MS System (2018 Samples)	API 5000 LC-MS/MS System (2020 Samples)	QSight LX50 LC-MS/MS System (2018 Samples)	API 5000 LC-MS/MS System (2020 Samples)
DON	0.130	0.313	0.420	1.042
DOM-1	0.136	0.217	0.450	0.725
NIV	0.018	0.150	0.060	0.500
AFM_1_	0.019	0.165	0.063	0.549
ZEN	0.001	0.006	0.003	0.020
α-ZOL	0.002	0.006	0.007	0.020
β-ZOL	0.008	0.006	0.025	0.019
FB_1_	0.005	0.147	0.017	0.490
FB_2_	0.005	0.658	0.016	2.193
OTA	0.0003	0.026	0.001	0.088
T-2	0.250	0.039	0.830	0.130
HT-2	6.250	0.046	20.800	0.152

Abbreviations: LOD, limit of detection; LOQ, limit of quantification; ZEN, zearalenone; DON, deoxynivalenol; DOM-1, de-epoxy-deoxynivalenol; FB_1_, fumonisin B_1_; FB_2_, fumonisin B_2_; OTA, ochratoxin A; α-ZOL, alpha-zearalenol; β-ZOL, beta-zearalenol; NIV, nivalenol; AFM_1_, aflatoxin M_1_.

**Table 10:** LC-MS/MS conditions for NIV, HT-2 and T-2 by MRM method.

Analyte	Precursorion	Q1 (*m*/*z*)	Q3 (*m*/*z*)	DP (V)	EP (V)	CE (V)	CXP (V)
NIV	[NIV + CH_3_COO]^−^	371.2	311.1	−23	−6	−19	−2
			281.0			−19	−2
			59.1 ^a^			−38	−6
HT-2	[HT-2 + NH_4_]^+^	442.4	215.2 ^a^	60	10	19	10
			245.3			16	
			263.5			19	
T-2	[T-2 + NH_4_]^+^	484.3	185.3	45	7	30	3
			215.2 ^a^				
			245.2				

^a^ Transitions used for quantitation; Q1: first quadrupole; Q3: third quadrupole; DP: declustering potential; EP: entrance potential; CE: collision energy; CXP: collision cell exit potential.

## Data Availability

The original contributions presented in this study are included in the article. Further inquiries can be directed to the corresponding author.

## Abbreviations

The following abbreviations are used in this manuscript:

AFB_1_	Aflatoxin B_1_
AFM_1_	Aflatoxin M_1_
α-ZOL	Alpha-zearalenol
β-ZOL	Beta-zearalenol
CIT	Citrinin
DON	Deoxynivalenol
DOM-_1_	Deepoxy-deoxynivalenol
DON-_3_-GlcA	Deoxynivalenol-_3_-glukuronid
DON-_15_-GlcA	Deoxynivalenol-_15_-glukuronid
DON-GlcA	Deoxynivalenol-glukuronid
DOM-GlcA	Deepoxy-deoxynivalenol-glukuronid
EDI	Estimated Daily Intake
FB1	Fumonisin B_1_
FB2	Fumonisin B_2_
FDR	false discovery rate
FUM	Fumonisins
JECFA	Joint FAO/WHO Expert Committee on Food Additives
LOD	Limit of Detection
LOQ	Limit of Quantification
NIV	Nivalenol
nr	not relevant (<LOD)
OTA	Ochratoxin A
PDI	Probable Daily Intake
TDI	Tolerable Daily Intake
ZEN	Zearalenone
